# Early treatment-related neutropenia predicts response to palbociclib

**DOI:** 10.1038/s41416-020-0967-7

**Published:** 2020-07-09

**Authors:** Nicholas P. McAndrew, Mark A. Dickson, Amy S. Clark, Andrea B. Troxel, Mark H. O’Hara, Christopher Colameco, Maryann Gallager, Kristi Gramlich, Kelly Zafman, David Vaughn, Gary K. Schwartz, Peter J. O’Dwyer, Angela DeMichele

**Affiliations:** 1 Perelman School of Medicine at the University of Pennsylvania, Philadelphia, PA USA; 2 Division of Hematology/Oncology, Department of Medicine, University of Pennsylvania, Philadelphia, PA USA; 3 Abramson Cancer Center, University of Pennsylvania, Philadelphia, PA USA; 4 Department of Medicine, Memorial Sloan-Kettering Cancer Center, New York, NY USA; 5 Weill Cornell Medical College, New York, NY USA; 6 Department of Population Health, NYU School of Medicine, New York, NY USA; 7 Herbert Irving Cancer Center, Columbia University School of Medicine, New York, NY USA; 8 Center for Clinical Epidemiology and Biostatistics, University of Pennsylvania, Philadelphia, PA USA

**Keywords:** Breast cancer, Predictive markers, Sarcoma, Cancer therapeutic resistance

## Abstract

**Background:**

Palbociclib is highly active in oestrogen-receptor positive (ER+) metastatic breast cancer, but neutropenia is dose limiting. The goal of this study was to determine whether early neutropenia is associated with disease response to single-agent palbociclib.

**Methods:**

Blood count and disease-response data were analysed from two Phase 2 clinical trials at different institutions using single-agent palbociclib: advanced solid tumours positive for retinoblastoma protein and advanced liposarcoma. The primary endpoint was PFS. The primary exposure variable was the nadir absolute neutrophil count (ANC) during the first two cycles of treatment.

**Results:**

One hundred and ninety-six patients (61 breast, 135 non-breast) were evaluated between the two trials. Development of any grade neutropenia was significantly associated with longer median PFS in both the breast cancer (HR 0.29, 95% CI 0.11–0.74, *p* = 0.010) and non-breast cancer (HR 0.57, 95% CI 0.38–0.85, *p* = 0.006) cohorts. Grade 3–4 neutropenia was significantly associated with prolonged PFS in the non-breast cohort (HR 0.57, 95% CI 0.38–0.85, *p* = 0.006) but not in the breast cohort (HR 0.87, 95% CI 0.51–1.47, *p* = 0.596). Multivariate analysis yielded similar results.

**Conclusions:**

Treatment-related neutropenia in the first two cycles was significantly and independently associated with prolonged PFS, suggesting that neutropenia may be a useful pharmacodynamic marker to guide individualised palbociclib dosing.

**Clinical trials registration information:**

Basket Trial: NCT01037790; Sarcoma Trial: NCT01209598.

## Background

Palbociclib is an orally active cyclin-dependent kinase (CDK) 4/6 inhibitor that has been practice-changing in the treatment of ER+ metastatic breast cancer.^1^ The PALOMA-2 and PALOMA-3 studies’ demonstration of improved progression-free survival (PFS) compared to endocrine therapy alone led to palbociclib being the first CDK 4/6 inhibitor to receive Food and Drug Administration (FDA) approval in combination with letrozole^2^ or fulvestrant^3^ in the metastatic, hormone-receptor positive (HR+) setting. Since then, two other CDK 4/6 inhibitors, ribociclib and abemaciclib, have also gained FDA approval for use in combination with endocrine therapy in advanced breast cancer based on improved PFS,^4–6^ with abemaciclib also being approved as a single agent based on the results of MONARCH 1.^7^ In addition, overall survival analyses of MONALEESA-7, MONALEESA-3, and MONARCH-2 suggest significantly improved overall survival with the addition of CDK 4/6 inhibition, with the results of PALOMA-3 suggesting a non-significant trend towards improved overall survival.^8–11^

Though generally well tolerated, the dose-limiting and most frequent adverse effect of these agents is neutropenia,^2,3,12^ resulting in frequent dose reductions and treatment interruptions.^12,13^ But despite grade 3–4 neutropenia rates of 62–66% seen in the PALOMA studies,^2,3^ infectious complications are rare.^12^ This is likely due to the mechanism that palbociclib exerts on haematopoietic cells. Palbociclib has been demonstrated in vitro to cause bone marrow suppression via cell-cycle arrest, which is reversible upon withdrawal of the medication.^14^ This is distinct from the marrow suppression caused by cytotoxic chemotherapy, which is the result of DNA damage and apoptosis.^14^ Importantly, palbociclib’s effect on retinoblastoma protein (Rb)-positive breast cancer cells is also mediated through cell-cycle arrest.^15^ We hypothesised that the degree of observed palbociclib-induced neutropenia that occurs with initiation of treatment is a reflection of the magnitude of effective systemic cell-cycle inhibition and would therefore predict drug activity and efficacy, regardless of tumour type. To test this hypothesis, we investigated whether neutropenia is associated with disease response in two Phase 2 clinical trials of single-agent palbociclib.

## Methods

The methods for the two clinical trials analysed in this study have been previously reported.^16–19^ Both studies were open-label, non-randomised, Phase 2 clinical trials of single-agent palbociclib in patients with advanced Rb-positive cancers. Briefly, NCT01037790 (the “Basket Trial”) was conducted at the University of Pennsylvania and enrolled adults with advanced/metastatic malignancies that were refractory to standard therapy or had no available standard-of-care options, including breast cancer, Kras- or BRAF-mutated colorectal cancer, oesophageal cancer, gastric cancer, cisplatin-refractory germ cell tumours, or any tumour type (excluding small cell lung cancer and retinoblastoma) positive for CCND1/CCND2 amplification or CDK 4/6 mutations. The CDK 4/6 mutation and CCND1/CCND2 amplification cohorts for the Basket Trial were still open to enrolment at the time of this manuscript’s preparation, but all disease-specific cohorts had completed accrual. All enrolled patients at the time of original data abstraction in January 2016 were included in the analysis. NCT01209598 (the “Sarcoma Trial”) was conducted at Memorial Sloan Kettering Cancer Center and enrolled patients with advanced well-differentiated/dedifferentiated liposarcoma.^17^ In both studies, patients were given single-agent palbociclib starting at 125 mg daily for 21 days, followed by 7 days off. Efficacy assessments were conducted every two cycles. Patients were maintained on study treatment until disease progression, unacceptable toxicity, or physician/patient decision to terminate participation.

Algorithms for dose modification and interruption were slightly different for each trial (Supplemental Fig. 1). In general, the dose modification scheme for the Basket Trial was designed to allow for more dose reductions based on varying levels of cytopenia than the Sarcoma Trial, which utilised treatment breaks rather than dose reductions upon development of neutropenia.

The primary endpoint considered for this analysis was PFS, which was defined as the time interval from Day 1/Cycle 1 of treatment initiation to radiographic progression, clinical progression (either before or after first planned scan), or death from any cause.^20^ The primary exposure variable characterising early neutropenia was the nadir absolute neutrophil count (ANC) during the first 2 cycles of treatment, which was defined as the lowest ANC at any point during the first 56 days after starting study drug. This exposure variable was chosen to limit immortal time bias and to capture the majority of neutropenic events prior to the first planned efficacy assessment. Nadir ANC was represented both as a continuous variable and as a categorical variable, according to grade of neutropenia (per Common Terminology Criteria for Adverse Events (CTCAE) version 3). During cycle one, blood counts were assessed at least once per week. Grade 1 neutropenia is defined in CTCAE v3 as an ANC below “the lower limit of normal” and 1.5 K/μL; the lower limit of normal for ANC used to define grade 1 neutropenia in this analysis was 1.8 K/μL.^21^ PFS curves were estimated using the method of Kaplan and Meier^22^ and compared using log-rank test. Multivariate Cox proportional hazards regression models^23^ were constructed manually using forward selection and confirmed using backward selection. Candidate variables potentially associated with PFS were Eastern Cooperative Oncology Group (ECOG) performance status, number of prior lines of systemic therapy (both chemotherapy and hormonal therapy), baseline ANC, race, sex, study site, body mass index (BMI), age, and chemotherapy as immediate prior line therapy. These variables were screened for inclusion in the model using a significance level of *p* < 0.2 in a univariate analysis and ultimately retained in the model using a criterion of *p* < 0.05. Statistical analyses were performed using the Stata 14 Statistics/Data Analysis software package (StataCorp, College Station, TX).

## Results

A total of 196 patients were enrolled between the two trials, with 137 patients enrolled in the Basket Trial and 59 patients enrolled in the Sarcoma Trial. Demographic characteristics stratified by breast cancer vs other tumour subtypes are shown in Table 1. Patients with breast cancer, comprising the largest disease cohort (*N* = 61), were all female and were more heavily pretreated compared to patients with other tumours. With respect to other demographic differences between the two trials, patients in the Basket Trial were more likely to have a higher baseline ECOG performance status, a higher number of prior lines of therapy, and were more likely to have chemotherapy as the immediate prior line of therapy (Table 2). A total of 186 patients, or 95% of the combined sample, discontinued the study for progressive disease (*N* = 185) or death from any cause (*N* = 1). Other reasons for discontinuing study treatment were patient decision (*N* = 4), toxicity (*N* = 3), and physician decision (*N* = 2). One patient remained on active treatment at the time of data cut-off in January 2016, at which time this patient had completed 55 cycles of therapy. The ten patients who did not experience disease progression during the study period were included in the survival analyses below and censored at withdrawal or last known follow-up.

**Table 1:** Demographics by tumour type.

Cancer type	Breast	Other
Total patients	61	135
Age in years, median (range)	58 (34–88)	55 (17–87)
Sex, *N* (%)^a^		
Female	61 (100)	48 (36)
Male	0 (0)	87 (64)
Race, *N* (%)		
White	56 (92)	113 (84)
Black	3 (5)	9 (7)
Asian/other	2 (3)	13 (10)
BMI, median (range)^b^	25 (19–47)	26 (16–48)
Baseline ECOG PS, *N* (%)		
0	40 (66)	82 (61)
1	21 (34)	53 (39)
# Prior lines of therapy, *N* (%)^a^		
0	0 (0)	24 (18)
1	3 (5)	49 (36)
2	4 (7)	23 (17)
≥3	54 (88)	39 (29)
Immediate prior line chemotherapy, *N* (%)		
Yes	35 (57)	86 (64)
No	26 (43)	49 (36)
Baseline ANC (K/μL), median (range)	4.0 (1.6–10.4)	4.4 (1.5–31.9)

^a^Fisher’s Exact *p* < 0.05 (Wilcoxon rank-sum used for age, BMI, and baseline ANC).

^b^Missing BMI values for two patients in Basket Trial (due to unrecorded height).

**Table 2:** Demographics by trial.

Trial	Basket Trial	Sarcoma Trial
Total patients	137	59
Tumour type, *N* (%)^a^		
Breast	61 (45%)	0 (0%)
Liposarcoma	1 (<1%)	59 (100%)
Germ cell	30 (22%)	0 (0%)
Colorectal	22 (16%)	0 (0%)
Oesophageal	12 (9%)	0 (0%)
Other^b^	11 (6%)	0 (0%)
Age in years, median (range)^a^	56 (17–88)	62 (35–87)
Gender, *N* (%)		
Female	80 (58%)	29 (49%)
Male	57 (42%)	30 (51%)
Race, *N* (%)		
White	122 (89%)	47 (79%)
Black	8 (6%)	4 (7%)
Asian/other	7 (5%)	8 (14%)
BMI, median (range)^c^	25.8 (16.0–47.1)	27.2 (17.5–48.0)
Baseline ECOG PS, *N* (%)^a^		
0	71 (52%)	51 (86%)
1	66 (48%)	8 (14%)
# Prior lines of therapy, *N* (%)^a^		
0	2 (1%)	22 (37%)
1	23 (17%)	29 (49%)
2	23 (17%)	4 (7%)
≥3	89 (65%)	4 (7%)
Immediate prior line chemotherapy, *N* (%)^a^		
Yes	38 (28%)	37 (63%)
No	99 (72%)	22 (37%)
Baseline ANC (K/μL), median (range)	4.2 (1.5–31.9)	4.1 (1.5–10.2)

^a^Fisher’s Exact *p* < 0.05 (Wilcoxon rank-sum used for age, BMI, and baseline ANC).

^b^Others are diagnoses representing <5% of all patients (adrenal, gastric, glioblastoma, renal cell, and thymus cancers).

^c^Missing BMI values for two patients in Basket Trial (due to unrecorded height).

In the first two cycles, 155 patients (79%) experienced grade 1–4 neutropenia, with 41 patients (21%) not experiencing neutropenia. Of those not experiencing neutropenia, 5 patients had breast cancer (8% of all breast patients), and 36 had non-breast cancers (27% of all non-breast patients). Table 3 examines the clinical factors that were significantly associated with both any grade and grade 3–4 neutropenia within the first two cycles. Women, lower baseline ANC, and breast cancer diagnoses had a significantly increased incidence of any grade and grade 3–4 neutropenia. Patients with an ECOG performance status of 0 had a significantly increased incidence of any grade neutropenia, though not grade 3–4 neutropenia.

**Table 3:** Risk of neutropenia in cycles 1–2 by clinical characteristics.

Patient characteristics, *N* (%)	No neutropenia (*n* = 41)	Any grade neutropenia (*n* = 155)	Odds ratio (95% CI)	*p* Value	Grade 0–2 NTP (*n* = 129)	Grade 3–4 NTP (*n* = 67)	Odds ratio (95% CI)	*p* Value
Age in years								
<50	13 (32%)	47 (30%)	—	0.9855	38 (29%)	22 (33%)	—	0.6806
50–69	21 (51%)	81 (52%)	1.07 (0.48–2.33)		70 (54%)	32 (48%)	0.79 (0.40–1.55)	
≥70	7 (17%)	27 (18%)	1.07 (0.38–3.00)		21 (16%)	13 (19%)	1.07 (0.45–2.55)	
Sex								
Male	26 (63%)	61 (39%)	—	0.0059	68 (53%)	19 (28%)	—	0.001
Female	15 (37%)	94 (61%)	2.67 (1.48–3.71)		61 (47%)	48 (72%)	2.82 (1.49–5.31)	
Race								
Black	1 (2%)	11 (7%)	—	0.4263	7 (5%)	5 (7%)	—	0.2264
White	36 (88%)	133 (86%)	0.33 (0.04–2.69)		115 (90%)	54 (81%)	0.66 (0.20–2.1)	
Other	4 (10%)	11 (7%)	0.25 (0.02–2.61)		7 (5%)	8 (12%)	1.60 (0.35–7.40)	
BMI^a^								
<Median	16 (40%)	80 (52%)	—	0.1768	59 (46%)	37 (56%)	—	0.188
≥Median	24 (60%)	74 (48%)	0.62 (0.30–1.25)		69 (54%)	29 (44%)	0.67 (0.37–1.22)	
ECOG performance status								
0	18 (44%)	104 (67%)	—	0.0072	76 (59%)	46 (67%)	—	0.1791
1	23 (56%)	51 (33%)	0.38 (0.19–0.77)		53 (41%)	21 (31%)	0.65 (0.35–1.22)	
# Prior lines of therapy								
0	6 (15%)	18 (12%)	—	0.5859	17 (13%)	7 (11%)	—	0.4799
1	9 (22%)	43 (28%)	1.59 (0.49–5.13)		37 (29%)	15 (22%)	0.98 (0.34–2.86)	
2	8 (19%)	19 (12%)	0.79 (0.22–2.73)		19 (15%)	8 (12%)	1.02 (0.31–3.42)	
≥3	18 (44%)	75 (48%)	1.39 (0.48–4.00)		56 (43%)	37 (55%)	1.60 (0.61–4.25)	
Immediate prior line chemotherapy								
No	30 (73%)	91 (59%)	—	0.0843	79 (61%)	42 (63%)	—	0.8432
Yes	11 (27%)	64 (41%)	1.91 (0.90–4.11)		50 (39%)	25 (37%)	0.94 (0.51–1.73)	
Baseline ANC								
<Median	4 (10%)	90 (58%)	—	<0.0001	44 (34%)	50 (75%)	—	<0.0001
≥Median	37 (90%)	65 (42%)	0.08 (0.03–0.23)		85 (66%)	17 (25%)	0.18 (0.09–0.34)	
Trial								
Basket Trial	32 (78%)	105 (68%)	—	0.1906	87 (67%)	50 (75%)	—	0.2942
Sarcoma Trial	9 (22%)	50 (32%)	1.69 (0.75–3.82)		42 (33%)	17 (25%)	0.70 (0.36–1.37)	
Breast cancer								
No	36 (88%)	99 (64%)	—	0.0017	99 (77%)	36 (53%)	—	0.0011
Yes	5 (12%)	56 (36%)	4.07 (1.51–10.97)		30 (23%)	31 (46%)	2.84 (1.51–5.34)	

*ANC* absolute neutrophil count, *BMI* body mass index, *ECOG* Eastern Cooperative Oncology Group, *NTP* neutropenia.

^a^Height missing from two patients (one patient with no NTP, one with grade 3 NTP).

**Fig. 1:**
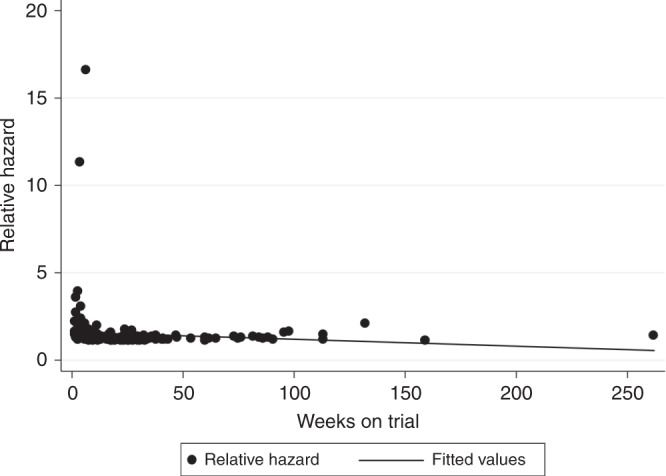
Nadir ANC in cycles 1–2 vs PFS weeks—Cox predicted relative hazard values. Spearman’s rho = −0.4073 (*p* < 0.001). HR 1.22 (95% CI 1.13–1.32).

**Fig. 2:**
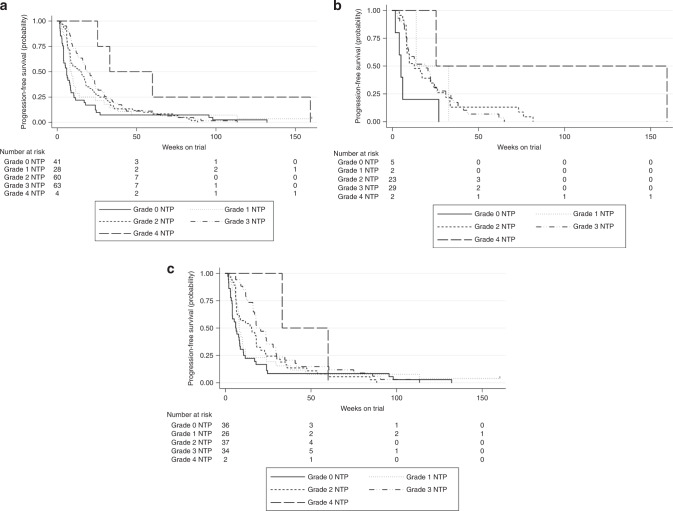
Kaplan-Meier survival by cycle 1–2 maximum grade neutropenia. **a** All patients. Grade 0: (reference); median PFS 6.0 weeks. Grade 1: HR 0.65 (0.40–1.07), *p* = 0.092; median PFS 8.1 weeks. Grade 2: HR 0.60 (0.40–0.90), *p* = 0.014; median PFS 15.6 weeks. Grade 3: HR 0.49 (0.33–0.74), *p* = 0.001; median PFS 19.4 weeks. Grade 4: HR 0.19 (0.06–0.62), *p* = 0.006; median PFS 60.0 weeks. Overall log-rank *p* = 0.002. **b** Breast patients. Grade 0: (reference); median PFS 5.0 weeks. Grade 1: HR 0.30 (0.05–1.56), *p* = 0.152; median PFS 13.9 weeks. Grade 2: HR 0.27 (0.10–0.75), *p* = 0.012; median PFS 16.3 weeks. Grade 3: HR 0.33 (0.12–0.87), *p* = 0.024; median PFS 19.4 weeks. Grade 4: HR 0.05 (0.01–0.52), *p* = 0.011; median PFS 25.4 weeks. Overall log-rank *p* = 0.028. **c** Non-breast patients. Grade 0: (reference); median PFS 6.0 weeks. Grade 1: HR 0.73 (0.43–1.23), *p* = 0.232; median PFS 8.0 weeks. Grade 2: HR 0.66 (0.41–1.06), *p* = 0.083; median PFS 15.6 weeks. Grade 3: HR 0.46 (0.28–0.75), *p* = 0.002; median PFS 18.0 weeks. Grade 4: HR 0.20 (0.02–1.17), *p* = 0.071; median PFS 60.0 weeks. Overall log-rank *p* = 0.013.

Figure 1 displays the relationship between each patient’s nadir ANC in the first two cycles (as a continuous variable) and PFS weeks as a predicted relative hazard based on a univariate Cox regression model. The Spearman’s rho of −0.41 (*p* < 0.001) indicates that decreasing values for nadir ANC were significantly associated with prolonged PFS weeks (hazard ratio (HR) 1.22, 95% confidence interval (CI) 1.13–1.32, *p* < 0.001). In the breast cancer subgroup, nadir ANC in cycle 1–2 (as a continuous variable) was also associated with PFS (HR 1.73, 95% CI 1.02–2.97, *p* = 0.042). The nadir ANC was also analysed as a categorical variable according to the maximum grade of neutropenia in the first two cycles. For all patients, increasing grade of neutropenia was associated with significantly prolonged median PFS (log-rank *p* = 0.002), with corresponding HR and median PFS weeks for each grade of neutropenia listed below in Fig. 2a. The relationship between increasing grade of neutropenia and prolonged PFS remained significant after analysing patients with breast (Fig. 2b, log-rank *p* = 0.028) and non-breast (Fig. 2c, log-rank *p* = 0.013) tumours separately.

**Fig. 3:**
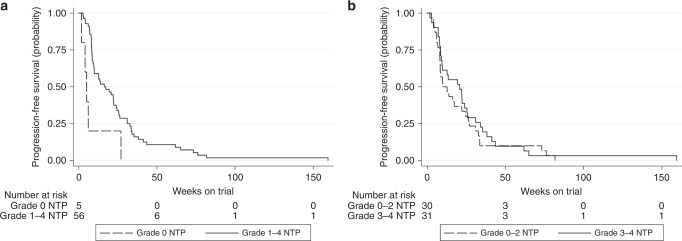
Kaplan-Meier survival by cycle 1–2 any grade or grade 3–4 neutropenia (breast patients). **a** Any grade neutropenia. Grade 0: (reference); median PFS 5.0 weeks. Grade 1–4: HR 0.29 (95% CI 0.11–0.74), *p* = 0.010; median PFS 17.3 weeks. **b** Grade 3–4 neutropenia. Grade 0–2: (reference); median PFS 12.7 weeks. Grade 3–4: HR 0.87 (95% CI 0.51–1.47), *p* = 0.596; median PFS 20.7 weeks.

Examining the patients with breast cancer in closer detail, those who experienced grade 1–4 neutropenia in the first two cycles had significantly prolonged PFS compared to those who experienced grade 0 neutropenia, with median PFS of 17.3 and 5 weeks, respectively (HR 0.29, 95% CI 0.11–0.74, *p* = 0.010, Fig. 3a). Nine patients who had HER2-positive disease also received concurrent trastuzumab. Excluding these patients from the above analysis resulted in similar findings (HR 0.32, 95% CI 0.12–0.82, *p* = 0.018). There was no difference in median PFS for breast cancer patients who experienced grade 3–4 neutropenia compared to those with grade 0–2 neutropenia (20.7 vs 12.7 weeks, respectively, HR 0.87, 95% CI 0.51–1.47, *p* = 0.596, Fig. 3b).

Non-breast cancer patients who experienced any grade neutropenia also had significantly prolonged PFS compared to those with grade 0 neutropenia (median PFS 16.1 vs 6 weeks, respectively, HR 0.57, 95% CI 0.38–0.85, *p* = 0.006). In addition, grade 3–4 neutropenia was significantly associated with prolonged PFS compared to grade 0–2 neutropenia in the non-breast group (median PFS 20.6 vs 9.5 weeks, respectively, HR 0.57, 95% CI 0.38–0.85, *p* = 0.006).

Multivariate Cox regression models were constructed for all patients, as well as both the breast and non-breast cohorts, and are shown in Table 4. The screening univariate Cox analysis is also provided in Supplementary Table 1. Using both forward and backward variable selection, any grade of neutropenia within the first two cycles, age (above the median value), and immediate prior line consisting of chemotherapy remained significantly associated with PFS in the breast group. In the non-breast group as well as the overall population, any grade of neutropenia within the first two cycles, ECOG performance status, and number of prior lines of therapy were significantly associated with PFS. Adjusting for these variables, any grade of neutropenia remained significantly and independently associated with prolonged PFS (overall: HR 0.49, 95% CI 0.34–0.70, *p* < 0.001; breast: HR 0.19, 95% CI 0.07–0.52, *p* = 0.001; non-breast: 0.56 95% CI 0.37–0.83, *p* = 0.004).

**Table 4:** Multivariate Cox regression analysis.

Covariate	HR (95% CI)	*p* Value
All patients		
Any NTP in C1–2		
No		
Yes	0.49 (0.34–0.70)	<0.001
ECOG PS		
0		
1	1.51 (1.12–2.04)	0.007
# Prior lines of therapy		
1–2		
3+	1.39 (1.02–1.87)	0.033
Breast patients		
Any NTP in C1–2		
No		
Yes	0.19 (0.07–0.52)	0.001
Age		
<Median		
≥Median	0.50 (0.29–0.89)	0.017
Immediate prior line chemo		
No		
Yes	1.76 (1.02–3.03)	0.042
Non-breast patients		
Any NTP in C1–2		
No		
Yes	0.56 (0.37–0.83)	0.004
ECOG PS		
0		
1	1.68 (1.15–2.43)	0.006
# Prior lines of therapy		
1–2		
3+	1.81 (1.22–2.70)	0.003

## Discussion

Neutropenia is the most important adverse effect of palbociclib because of both its high frequency and impact on drug dosing. We found that neutropenia following single-agent palbociclib was strongly associated with higher PFS across a variety of diseases and was independent of other clinical factors. The strong association of early neutropenia with PFS suggests that the neutrophil count in cycles 1 and 2 may predict likelihood of disease response. Because patients remained on trial until clinical or radiographic progression, these findings also support improved duration of response.

Our findings confirm and expand on analyses of the PALOMA-3 trial in breast cancer in which PFS was also examined in relation to neutrophil count and dose-reduction status.^24^ In PALOMA-3, grade 3 or 4 neutropenia was not associated with prolonged PFS in the experimental group, a finding confirmed in the present analysis. However, our data suggest that early grade 0 neutropenia may predict reduced response to palbociclib. While a “dose effect” association of decreasing nadir ANC and increasing median PFS was observed (Figs. 1 and 2a, b), grades 2 and 3 neutropenia represented the majority of maximum cycle 1–2 grade neutropenia in the breast cohort. The predominance of grades 2 and 3 neutropenia is the likely explanation for no observed difference in PFS between grade 3–4 and grade 0–2 neutropenia in the breast cohort. This may also be the reason why the data from PALOMA-3 do not suggest an association between neutropenia and PFS, as only grade 3–4 vs grade 0–2 neutropenia was analysed in this trial.^24^ The improved PFS with grade 3–4 neutropenia in the non-breast cohort was likely driven by the significantly increased proportion of patients with grade 0 neutropenia compared to the breast cohort.

It is important to recognise that while patients experiencing neutropenia within the first two cycles had longer PFS, it is possible that patients who did not experience neutropenia might still have received some benefit from the drug. Both studies were single-arm trials, therefore there is no control arm against which the PFS for the grade 0 neutropenia group can be compared. The basis for favourable outcomes in patients with some degree of toxicity is potentially suggested by previous studies of neutropenia in Phase 2 and Phase 2 data sets. Sun et al.^25^ used a population pharmacokinetic–pharmacodynamic model to study the relationship between drug levels and ANC by combining data from three different clinical trials that studied palbociclib either alone or in combination with letrozole. They found that higher levels of palbociclib exposure were associated with lower ANC values and that this neutropenia was rapidly reversible and noncumulative. In addition, there is further preclinical evidence from Hu et al.^14^ of a dose–response relationship between increasing palbociclib dose and increasing levels of neutropenia. Furthermore, Im et al. performed an analysis of the PALOMA-2 data in Asian women and found both higher rates of neutropenia as well as higher palbociclib trough concentrations compared to non-Asians.^26^ Conversely, Iwata et al.^27^ reported safety and efficacy data from PALOMA-3 comparing rates of neutropenia, relative dose intensity, and palbociclib trough concentrations between Asian and non-Asian patients. While patients in the Asian arm had higher rates of neutropenia and required more dose reductions for neutropenia than non-Asian patients, the palbociclib trough concentrations were not significantly different between the two groups.

The fact that our study utilised data from a variety of malignancies and included two partner studies that handled dose modification in different ways are particular strengths, suggesting that this is not a disease-specific effect but rather a pharmacodynamic one. The overall sample size, as well as relatively higher numbers of patients with breast cancer and liposarcoma, provides adequate power to detect the HRs in both the overall group and specific disease subsets, as described above. In addition, the multivariate models constructed strongly suggest that this association is independent of other clinical factors. However, patients with other malignancies are represented in smaller numbers, and careful consideration should be taken when applying findings to these patients. Also, it is important to acknowledge that many patients with early clinical progression will contribute shorter PFS times, therefore these results should be interpreted cautiously, and further confirmation of these findings is needed. Finally, the lack of pharmacokinetic data is a limitation of this study, preventing any investigation of the association between circulating drug levels and serum neutropenia, and should be incorporated into future studies of this relationship.

## Conclusions

Patients who experienced palbociclib-related neutropenia in the first two cycles of treatment had significantly improved PFS compared to patients who did not experience neutropenia. This effect was seen across multiple tumour types, including breast cancer, and remained significant in a multivariate analysis. Even though patients experiencing grade 0 neutropenia exhibited reduced PFS relative to those experiencing any grade neutropenia, it is still possible that they received some benefit from the drug. These findings, validating preclinical data and the clinical findings in PALOMA-3, have the potential to impact clinical decision-making and future palbociclib development. Future research is needed to better understand the underlying mechanism of this effect and, if confirmed, whether it can be exploited to optimise therapeutic effect by matching an individual’s palbociclib dose to a target ANC. A prospective trial comparing flat dosing to dosing tailored to a target ANC is warranted to determine whether neutropenia could be clinically utilised as a predictive biomarker and identify patients who may be under-dosed and not achieving effective cell-cycle inhibition with the FDA-approved dose.

## Supplementary information

Supplemental Table 1 (A-C) and Supplemental Figure 1 (A-B)

## Data Availability

The data analysed in this study are securely managed by the study team and not available as part of a public database but can be made available in a deidentified fashion if requested by the editorial board.
