# Supplementary material for: Early treatment-related neutropenia predicts response to palbociclib
Source: Br J Cancer. 2020 Jul 9;123(6):912–8. doi: 10.1038/s41416-020-0967-7 (PMC7492243; doi:10.1038/s41416-020-0967-7)
Supplement: Supplementary file 1 — Supplemental Table 1 (A-C) and Supplemental Figure 1 (A-B) [file 41416_2020_967_MOESM1_ESM.docx]

**Supplemental Material**

Supplementary Figure 1: Dose Modification Schemes (Online Only)

Supplementary Figure 1a: Dose Modification Scheme for Basket Trial


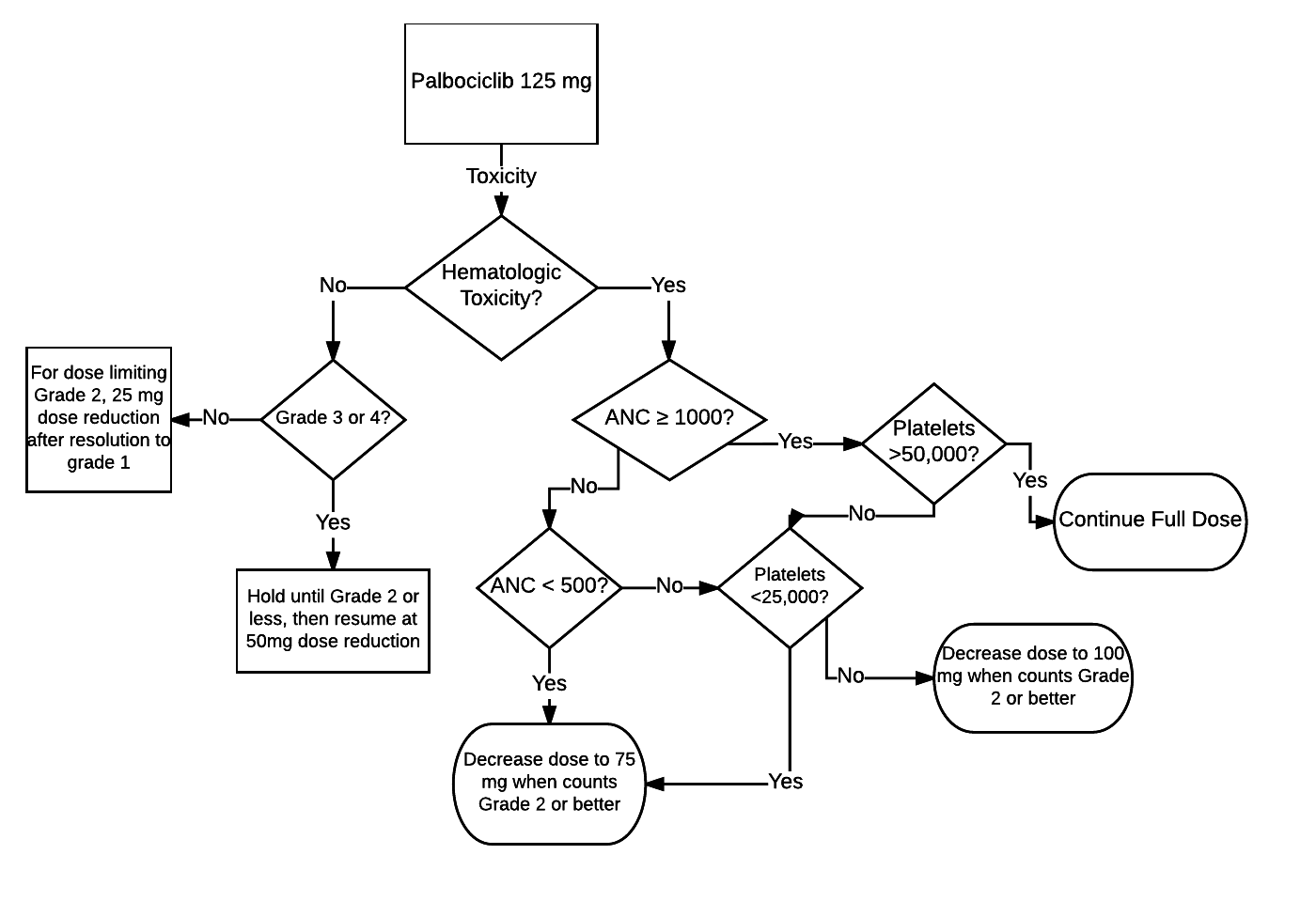


Supplementary Figure 1b: Dose Modification Scheme for Sarcoma Trial


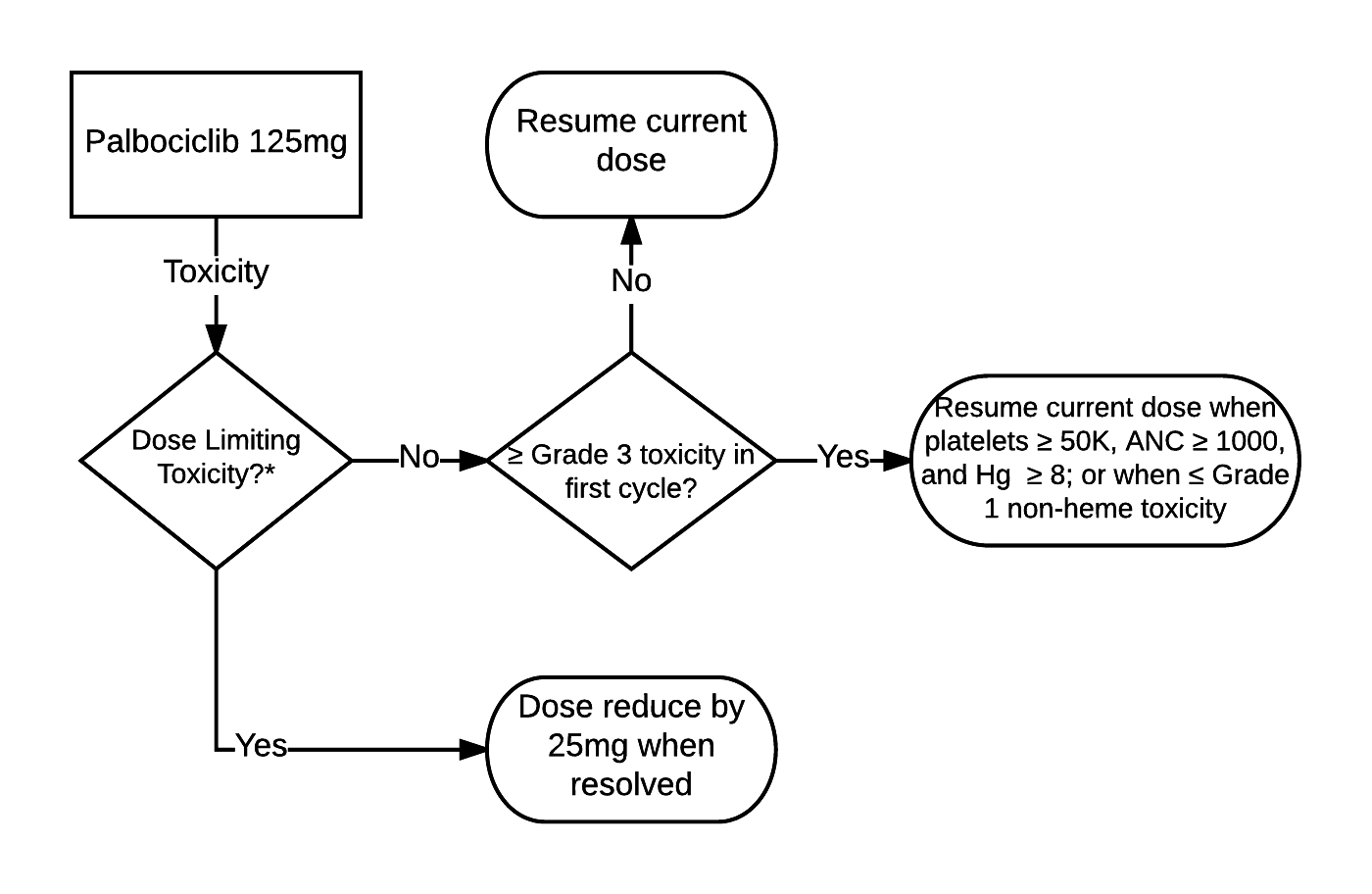


*DLT = Grade 4 hematologic toxicity, ANC < 1,000/μL with associated fever, ≥ Grade 3 non-hematologic treatment related toxicity (that have otherwise been maximally treated), lack of hematologic recovery to < Grade 3 (platelets <50,000/μL, ANC <1,000/μL, and hemoglobin <8.0 g/dL) 1 week after last cycle

ANC = Absolute Neutrophil Count; Hg = Hemoglobin

Supplementary Table 1a – Screening Univariate Cox Regression Analysis (All Patients)

| Covariate | HR (95% CI) | p value |
| --- | --- | --- |
| Any NTP in C1-2  No  Yes | 0.54 (0.37-0.76) | 0.001 |
| ECOG PS  0  1 | 1.54 (1.14-2.08) | 0.004 |
| # Prior Lines of Therapy  1-2  3+ | 1.32 (0.99-1.77) | 0.060 |
| Baseline ANC  < median  ≥ median | 1.30 (0.97-1.73) | 0.077 |
| Race  Non-White  White | 1.53 (1.01-2.31) | 0.043 |
| Sex  Male  Female | 0.94 (0.71-1.27) | 0.703 |
| Trial  Basket  Sarcoma | 0.79 (0.57-1.08) | 0.140 |
| BMI  < median  ≥ median | 0.83 (0.62-1.11) | 0.200 |
| Age  < median  ≥ median | 1.03 (0.77-1.37) | 0.851 |
| Immediate Prior Line Chemo  No  Yes | 1.07 (0.80-1.44) | 0.656 |

Supplementary Table 1b – Screening Univariate Cox Regression Analysis (Breast Patients)

| Covariate | HR (95% CI) | p value |
| --- | --- | --- |
| Any NTP in C1-2  No  Yes | 0.29 (0.11-0.74) | 0.010 |
| ECOG PS  0  1 | 1.02 (0.57-1.81) | 0.941 |
| # Prior Lines of Therapy  1-2  3+ | 1.13 (0.50-2.57) | 0.771 |
| Baseline ANC  < median  ≥ median | 1.07 (0.63-1.81) | 0.810 |
| Race  Non-White  White | 2.43 (0.87-6.80) | 0.092 |
| Sex  Male  Female | - | - |
| Trial  Basket  Sarcoma | - | - |
| BMI  < median  ≥ median | 0.92 (0.54-1.57) | 0.763 |
| Age  < median  ≥ median | 0.61 (0.35-1.05) | 0.072 |
| Immediate Prior Line Chemo  No  Yes | 1.44 (0.85-2.43) | 0.171 |

Supplementary Table 1c – Screening Univariate Cox Regression Analysis (Non-Breast Patients)

| Covariate | HR (95% CI) | p value |
| --- | --- | --- |
| Any NTP in C1-2  No  Yes | 0.57 (0.39-0.85) | 0.006 |
| ECOG PS  0  1 | 1.82 (1.27-2.62) | 0.001 |
| # Prior Lines of Therapy  1-2  3+ | 1.92 (1.30-2.83) | 0.001 |
| Baseline ANC  < median  ≥ median | 1.43 (1.004-2.02) | 0.047 |
| Race  Non-White  White | 1.40 (0.88-2.22) | 0.152 |
| Sex  Male  Female | 0.97 (0.67-1.40) | 0.876 |
| Trial  Basket  Sarcoma | 0.68 (0.48-0.97) | 0.035 |
| BMI  < median  ≥ median | 0.76 (0.53-1.07) | 0.117 |
| Age  < median  ≥ median | 1.22 (0.86-1.73) | 0.271 |
| Immediate Prior Line Chemo  No  Yes | 0.93 (0.65-1.34) | 0.696 |
